# Society Meetings

**Published:** 1908-08

**Authors:** 


### SOCIETY MEETINGS.

The American Association of Obstetricians and Gynecologists will hold its twenty-first annual meeting at the Hotel Belvedere, Chase and Charles Streets, Baltimore, Md., Tuesday, Wednesday and Thursday, September 22, 23 and 24, 1908, under the presidency of Dr. E. Gustav Zinke, of Cincinnati. Drs. W. A. B. Sellman, Joseph H. Branham, and William S. Smith, of Baltimore, constitute the committee of arrangements.

The following is a list of papers offered up to July 25, 1908.

1. The president's address, The solving of the problem of obstetrics, E. Gustav Zinke, Cincinnati.
2. Pubiotomy on the non-pregnant woman; report of a case operated, John N. Bell, Detroit.
3. The technic of the operation for periappendiceal abscess, William A. B. Sellman, Baltimore.
4. The comparative merits of abdominal celiotomy and colpotomy in the treatment of intrapelvic abscess, William S. Smith, Baltimore.
5. Shock in obstetrical practice, Adam H. Wright, Toronto.
6. Fibroid tumor of the uterus resembling pregnancy, with exhibition of specimen, Raleigh R. Huggins, Pittsburg.
7. Report of a case of impossible labor from ovarian dermoid; Cesarean section; recovery. Induction of labor in the second confinement, Walter P. Manton, Detroit.
8. Ovarian cystomata complicating pregnancy, Charles G. Cumston, Boston.
9. Experiments upon animals relative to the question of abdominal supporters after laparotomy, Robert T. Morris, New York.
10. A short history of fibroid operations during pregnancy, J. H. Carstens, Detroit.

11. Subdiaphragmatic abscess with report of cases, John W. Keefe, Providence.

12. Specimen of uterus and appendages showing a recent curet perforation and the result of vaginal section for extrauterine pregnancy seven years before hysterectomy, Joseph H. Branham, Baltimore.

13. Intraligamentous fibroids, John F. Erdmann, New York.

14. Advanced ectopic gestation with living child, with report of three cases, X. O. Werder, Pittsburg.

15. Extrauterine pregnancy, H. E. Hayd, Buffalo.

16. Ectopic gestation, Charles L. Bonifield, Cincinnati.

17. Some old fallacies in retroversion surgery revived, Albert Goldspohn, Chicago.

18. Treatment of typhoid fever perforation, John D. S. Davis, Birmingham.

19. Placenta succenturiata, with report of two cases, Edward T. Abrams, Dollar Bay.

20. Repair rather than removal of the internal generative organs of women, J. Egerton Cannaday, Wheeling, W. Va.

21. Factors of safety in abdominal operations, based on operations, George W. Crile, Cleveland.

22. A simple, certain and universally applicable method of preventing the serious accident of leaving a sponge in the abdomen, H. S. Crossen, Saint Louis.

23. Acute pancreatitis, with report of case, Louis Frank, Louisville.

24. Postoperative hiccough, Edward T. Abrams, Dollar Bay.

25. Removal of fibroid tumor from the pregnant uterus. William J. Gillette, Toledo.

26. Disorders of the female bladder, Ramon Guiteras, New York.

27. My experience with the Gilliam operation, William H. Humiston, Cleveland.

28. Typhlitis, John A. Lyons, Chicago.

29. Remarks on myoma of the cervix uteri, with presentation of specimen, Francis Reder, Saint Louis.

30. Arteriosclerosis of the uterus, Charles M. Rees, Charleston.

31. Injuries to the bladder occurring during hernia operations, Roland E. Skeel, Cleveland.

32. Abscess of Gaertner's canal, with report of a case, Magnus A. Tate, Cincinnati.

33. Hysteria as the surgeon sees it, Ap. Morgan Vance, Louisville.

34. History and treatment of uterine fibroids, A. Vander Veer, Albany.

35. Fallacy of purgation, Edwin Walker, Evansville.

The following named Fellows have promised papers the titles of which have not been supplied as yet: John Young Brown, Saint Louis; Henry Schwarz, Saint Louis; Frederick Blume, Pittsburg; Walter B. Dorsett, Saint Louis; Thomas B. Eastman, Indianapolis; Carlton C. Frederick, Buffalo; H. S. Griffin, Hamilton, Ont.; W. D. Haggard, Nashville; Rufus B. Hall, Cincinnati; C. S. Hamilton, Columbus; H. W. Longyear, Detroit; L. C. Morris, Birmingham; John B. Murphy, Chicago; L. S. McMurtry, Louisville; H. O. Pantzer, Indianapolis; Joseph Price, Philadelphia; Charles A. L. Reed, Cincinnati; M. Rosenwasser, Cleveland; F. F. Simpson, Pittsburg; Sigmar Stark, Cincinnati; William H. Wenning, Cincinnati.

